# Supplementary material for: Chance elections, social distancing restrictions, and KENTUCKY’s early COVID-19 experience
Source: PLoS One. 2021 Jul 1;16(7):e0250152. doi: 10.1371/journal.pone.0250152 (PMC8248727; doi:10.1371/journal.pone.0250152)
Supplement: Attachment — Submitted filename: Referee Report_KY.pdf [file pone.0250152.s002.pdf]

**Report: "Did Social-Distancing Measures in Kentucky Help to Flatten the COVID-19 Curve?"**

*Overview*

Emerging literatures in economics and public health find that state and local shelter-in-place orders (SIPOs) were effective in curbing COVID-19 case growth, particularly if enacted early in the outbreak cycle. This study contributes to these literatures with a case study of Kentucky. Specifically, the study explores the comparative effectiveness of a series of policies adopted in Kentucky to fight COVID-19 spread: public school closures, restaurant dining room closures, entertainment center closures, non-essential business closures, and a "Healthy at Home" (HAH) order. Event-study analyses show that the HAH order was most effective at curbing COVID-19, followed by restaurant/ entertainment center closure. The authors estimate that over 40,000 COVID-19 cases were averted in Kentucky due to social distancing policies adopted.

*Comments*

1. Given that there is substantial heterogeneity in both (i) state and local social distancing policies, and (ii) responses to such policies (due to differences in local populations), I believe that there is strong value-added in case studies of particular state (or local) policies. Therefore, it does not bother me that other published studies (including one by this team) have pooled Kentucky with other states/counties and estimated COVID-19 case growth effects of social distancing policies.

However, the authors could do a better job of making the case for "Why Kentucky?" Is there something unique about the policies (i.e., the substance of the orders, the timing of the orders, the time between each order, the legal enforceability of the orders?) in Kentucky compared to other states that makes them especially interesting? Is there something unique about the population that could generate unique policy responses? Making this case forcefully in the Introduction would help convince readers of the paper's contribution relative to the existing literature.

2. The research design of this study surprised me a little. Given the title, I was expecting the authors to "directly" estimate the effect of Kentucky's social distancing policies on COVID-19 case growth in Kentucky. Instead, the authors pool Kentucky with other states (as part of two census regions), estimate average treatment effects (across all jurisdictions in these regions) in an event-study framework, and then use these estimates to infer Kentucky's COVID-19 case avoidance. (This approach is very similar to the approach taken by Courtemanche et al. 2020 in their *Health Affairs* paper – the main difference is that here, the authors obtain estimates of social distancing policy impacts using a restricted sample of the Midwest and South Census Regions.)

My question is: why not directly estimate the effect of Kentucky's policies? For example, why not estimate the effects of KY's HAH order using, for example, a synthetic control design? The other social distancing policies could be accounted for by "matching" on the share of days over the sample period that the policies were in effect.

Identifying donor states should be straightforward for the HAH policy (non-SIPO adopters and states that adopted SIPOs at least X days (5? 7?) after Kentucky's order. I concede that finding credible donors for some of the other policies may be difficult. For example, donors for the "public school closure" policy evaluation would have to rely on states that closed schools late given that these closures were nearly universal. Local policies create another complication. But a synthetic control approach seems like the most obvious research design to employ first. Relatedly, the authors could estimate a difference-in-difference (or event study) model using a sample consisting of Kentucky (the one treatment state) and the donor states. If synthetic controls are not an option to study Kentucky's policies – perhaps because the donor pool is too limited and not credible — this could be explained in a lengthy footnote so readers know this was considered and rejected.

A second advantage of pursuing an alternative estimation strategy (if it worked out!) is that it would make the value-added of the study larger. It would avoid the problem of this paper being seen as a "small tinkering" of the *Health Affairs* paper.

3. Is the outcome in this paper the same as the outcome in the *Health Affairs* paper? In the *HA* paper, the authors write:

> "The daily exponential growth rate was calculated as the natural log of cumulative daily COVID-19 cases minus the log of cumulative daily COVID-19 cases on the prior day." (p. 1238)

In this paper, the outcome is described as:

> "We use these data to compute each county's daily exponential growth rate in confirmed COVID-19 cases, which is equal to the natural log of daily COVID-19 cases minus the log of daily COVID-19 cases on the prior day." (p. 6)

Why the change from growth in cumulative daily cases to growth in daily cases? A case can be made for looking at either margin (growth in day to day cumulative cases vs day to day daily cases)…both are interesting. Readers should just understand the comparability or non-comparability of the estimates across these two papers. Or maybe it was just a typo?

4. The authors might mention information as an important channel through which some of these policies could affect social distancing and COVID-19 case growth. For instance, a HAH order may send important information to residents about the seriousness of the epidemic (or scare the heck out of people), which could increase stay-at-home behavior. Staying-at-home may also facilitate information gathering by watching more news reports (i.e. Daily White House Coronavirus Briefings, etc.)

5. Finally, the authors claim that in the absence of social distancing policies, COVID-19 cases in Kentucky would be an order of magnitude (11 to 12 times) higher. This a is a very large number and one that is likely to draw the attention of those skeptical of large

COVID case effects of SIPOs. As the authors know, there is a recent set of studies (Cronin and Evans 2020; Goolsbee and Syverson 2020; Gupta et al. 2020; Sears et al. 2020) that emphasize that most of the variation in social distancing behavior comes from "private responses" to information/risk/beliefs rather than responses to SIPOs. (Still, plenty of credible evidence shows that SIPOs have an effect over and above these private responses.) However, given this set of papers, the authors might want to frame their effect sizes in terms of what we know about (i) social distancing elasticities with respect to the policies they study, and (ii) plausible COVID-19 case elasticities with respect to social distancing. This comment is NOT meant as an instruction to reconcile this paper's findings with the above set of papers. Rather, the comment is designed to help the authors frame their magnitudes in the context of this growing literature.
