# Supplementary material for: Chance elections, social distancing restrictions, and KENTUCKY’s early COVID-19 experience
Source: PLoS One. 2021 Jul 1;16(7):e0250152. doi: 10.1371/journal.pone.0250152 (PMC8248727; doi:10.1371/journal.pone.0250152)
Supplement: Attachment — Submitted filename: Response to Reviewers 1.docx [file pone.0250152.s003.docx]

Gatton College of

Business and Economics

Department of Economics

February 16, 2021

Dear Dr. Picone,

Thank you for the opportunity to revise and resubmit our manuscript for possible publication in PLOS One. We feel that the paper has improved considerably after revising it to address the referees’ comments, and we hope you agree. In our “response to reviewers” document (uploaded separately), we provide point-by-point responses to each comment. Below we highlight the most important changes.

The primary critique of both reviewers was that the contribution of the paper was modest in light of our paper published in *Health Affairs* in May, which studied the impacts of the same social distancing restrictions across the entire U.S. Effectively, the only difference in the analyses between that paper and the initial version of this one was the restriction of the sample to the Midwest and South, with the motivation being to obtain results that were relevant to relatively rural, politically conservative states like Kentucky.

In the revised version of the paper, we have sharpened this contribution in a way that should make the novelty much more clear:

(1) We have rewritten much of the introduction to better articulate *why* it is important to obtain results specific to right-leaning, relatively rural, predominantly “white working class” states like Kentucky. In other words, why can we not just assume results obtained using a nationwide sample are applicable? In short, (i) shelter-in-place orders (SIPOs) are effectively unenforceable nudges that rely on social pressure, so underlying attitudes about their appropriateness likely influence compliance; (ii) restaurant and entertainment establishment closures might have less of an impact on small town “working class” communities simply because such establishments are less prevalent there.
(2) We have added a new empirical analysis to capture this heterogeneity more directly. We developed a county-level “white working-class” index that is based on the county’s population density and percentages of residents who are white, Evangelical Christian, voted for President Trump, and do not have college degrees. We then re-estimate the regression model adding interactions of this index with each of the policy variables. The results show that the effectiveness of social distancing restrictions decreases markedly with higher values of the index. The results imply that the restrictions did little to flatten the curve in counties that are one standard deviation more “white working class” than average. We feel that this result adds important nuance to a literature that currently, on balance, argues that SIPOs and restaurant/entertainment facility closures were quite effective.

A related but distinct critique is why the particular emphasis on Kentucky? We have added new exposition and analyses to better make the case that Kentucky’s early COVID-19 experience was interesting and important.:

(1) The abstract and introduction now explain how a unique set of circumstances made Kentucky “as good as randomly” assigned to relatively strong, early restrictions. The confluence of a uniquely unpopular Republican incumbent governor and the Democratic challenger being the son of a former governor led to the Democrat winning the 2019 gubernatorial race in a “coin-flip” election in an otherwise deeply red state.
(2) The abstract and introduction now also explains that Kentucky is heavily “white working class” even after restricting the sample to the Midwest and South (its index value is roughly one standard deviation above the average for those regions). Therefore, the arguments about heterogeneity made above – and corresponding regression that adds interactions of the index with the policy variables – are particularly relevant to Kentucky.
(3) We have added simulations that show how the interaction effects imply that Kentucky’s experience with COVID-19 restrictions was much different in the two biggest cities of Louisville and Lexington than in the rest of the state. Specifically, we show that the restrictions flattened the COVID-19 curves substantially in Louisville and Lexington, given their below-average “white working-class” index values. However, the restrictions had little to no effect in the other counties of the state, which all have above-average index values.

Related to the emphasis on Kentucky, Reviewer 2 was surprised that we did not utilize a comparative case study method like synthetic control designed for situations with a single treated unit. We now explain in the paper why this is not feasible in this particular context. In short, synthetic control methods are not designed for multiple interventions implemented at multiple points in time in multiple treatment locations. That said, we agreed with the referee’s broader point that our empirical model should more directly connect to Kentucky if we wish to focus on that state for motivation and illustration. This is what led us to the “white working-class” index, as it enables estimates that are more directly applicable to Kentucky while still being econometrically feasible for a staggered, multiple-treatment context.

In addition to the referee’s comments, we also felt some revisions were necessary in light of the numerous developments in the course of the pandemic and the academic literature on it since our initial submission. First, we have updated our references to include new causally interpretable studies on the effectiveness of social distancing restrictions. Second, the last paragraph of the conclusion argues that the results are still important in the current phase of the pandemic.

Thank you again for your continued consideration and we look forward to your decision.

Sincerely,

Charles Courtemanche, on behalf of co-authors Joseph Garuccio, Anh Le, Josh Pinkston, and Aaron Yelowitz
